# Resting-State Sensory-Motor Connectivity between Hand and Mouth as a Neural Marker of Socioeconomic Disadvantage, Psychosocial Stress, Cognitive Difficulties, Impulsivity, Depression, and Substance Use in Children

**DOI:** 10.31586/jcn.2025.1280

**Published:** 2025-03-25

**Authors:** Shervin Assari, Alexandra Donovan, Babak Najand, Golnoush Akhlaghipour, Mario F Mendez

**Affiliations:** 1 Department of Internal Medicine, Charles R Drew University of Medicine and Science, Los Angeles, CA, USA; 2 Marginalized-Related Diminished Returns (MDRs) Research Center, Los Angeles, CA, USA; 3 Department of Urban Public Health, Charles R Drew University of Medicine and Science, Los Angeles, CA, USA; 4 Paul R. Korey Department of Neurology, Montefiore Medical Center, Bronx, NY, USA; 5 Department of Neurology, University of California Los Angeles (UCLA), Los Angeles, CA, USA; 6 Department of Psychiatry & Biobehavioral Sciences, University of California Los Angeles (UCLA), Los Angeles, CA, USA

**Keywords:** Sensory-Motor, Substance Use, Socioeconomic Status, Stress, Brain Connectivity

## Abstract

**Background:**

The sensory-motor network is essential for integrating sensory input with motor function and higher-order cognition. Resting-state functional connectivity (rsFC) within this network undergoes significant developmental changes, and disruptions in these connections have been linked to behavioral and psychiatric outcomes. However, the relationship between sensory-motor connectivity, early-life adversity, and later health behaviors remains understudied.

**Objective:**

This study examines the associations between rsFC within the sensory-motor network (mouth and hand regions) and key social, psychological, and behavioral factors, including baseline and past socioeconomic status (SES), trauma exposure, family conflict, impulsivity, major depressive disorder (MDD), and future substance use.

**Methods:**

Data were drawn from the Adolescent Brain Cognitive Development (ABCD) Study, a national sample of U.S. children. Resting-state fMRI data were used to assess functional connectivity within the sensory-motor network. Bivariate analyses examined associations between rsFC in the sensory-motor mouth and hand regions and baseline SES, past SES, childhood trauma exposure, family conflict, impulsivity, and MDD. Longitudinal analyses assessed whether baseline rsFC predicted future substance use.

**Results:**

Greater rsFC between the sensory-motor mouth and hand regions was significantly associated with lower SES, higher trauma exposure, and greater family conflict. Increased connectivity was also correlated with older age and more advanced puberty status. Higher rsFC between the sensory-motor mouth and hand regions was linked to greater impulsivity, lower cognitive function, an increased likelihood of MDD, and future marijuana use.

**Conclusion:**

These findings suggest that sensory-motor connectivity is sensitive to socioeconomic and psychosocial stressors, with potential long-term implications for mental health and substance use risk. The results highlight the importance of early-life environmental factors in shaping neurodevelopmental trajectories and emphasize the need for targeted interventions to mitigate the effects of adversity on brain function and behavior. Future research should further explore the role of sensory-motor network alterations in behavioral health outcomes as a function of environmental stressors.

## 1. Background

Sensory-motor network resting state connectivity is involved in various cognitive functions [1] including skill acquisition among both children and adults [2,3]. This network is also associated with drug use 4 and is altered in certain neurological disorders such as autism [5], cervical dystonia [6], and multiple sclerosis (MS) [7]. Recent literature suggests that sensory-motor networks play a crucial role in brain development, with potential implications for mental health and behavioral regulation [8]. In this view, sensory-motor connectivity may serve as a fundamental marker of neurodevelopment, reflecting maturation via the brain’s ability to integrate sensory input with motor output [8]. Alterations in sensory-motor brain network connectivity might serve as early biomarkers for psychopathology or maladaptive behaviors, which could inform more targeted prevention and intervention strategies [9-11].

The sensory-motor system is among the first neural circuits to develop, laying the foundation for movement, perception, and interaction with the external world. During infancy and early childhood, strong functional connectivity within sensory-motor circuits facilitate the coordination of basic movements, such as grasping, reaching, and oromotor functions like sucking and speech development [8]. The ability to control these movements becomes more precise, aided by the progressive integration of sensory-motor networks with cognitive and executive function regions. As the brain matures, connections to higher-order association cortices become more refined [12], supporting the transition from reflexive motor actions to deliberate, goal-directed behavior. This shift reflects the brain’s increasing capacity to regulate motor output based on complex decision-making, social interactions, and environmental demands [8].

Beyond its role in motor function, sensory-motor connectivity may also influence emotional and social development in children and adolescents. Sensorimotor circuits contribute to the body's perception of internal and external stimuli [13-16], which may influence emotional regulation [17-20] and stress responses [20,21]. In the literature, altered function of connectivity in these regions has been associated with depression, impulsivity, trauma-related hyperarousal, and somatic symptoms [9,21-29]. Therefore, the sensory-motor network may not only be involved in physical movement but also contributes to how individuals process emotions and respond to their environment. Disruptions in this process—potentially due to high environmental stressors or low socioeconomic resources—may contribute to impulsivity, difficulties with self-regulation, or susceptibility to maladaptive behaviors such as substance use [30-37]. Stronger-than-expected sensory-motor connectivity could indicate less integration with higher-order control networks (i.e., stronger intra connections commonly means weaker inter connections) [38-41], potentially making motor behaviors more automatic and less adaptable to cognitive control. Thus, sensory-motor connectivity may reflect the dynamic interplay between neural maturation and socioenvironmental influences (e.g., SES and stress), with significant implications for a wide range of cognitive functions and behavioral adaptations, including substance use [8].

Changes in sensory-motor connectivity may reflect adaptations to early-life stress, socioeconomic disadvantage, or psychiatric vulnerabilities, ultimately shaping cognitive and emotional function [42,43]. Increased connectivity could indicate hyperactivity in reflexive motor pathways, whereas reduced integration with association cortices may suggest weaker top-down cognitive control [44]. Understanding these connectivity patterns more deeply could offer valuable insights into neurodevelopmental disorders, psychiatric conditions, and the long-term effects of early-life adversity. Such knowledge could help inform targeted interventions to support healthy brain maturation. Therefore, it is essential to study the intersections of sensory-motor connectivity changes, socioeconomic status, stress, brain development, cognition, and behavior.

One example of this interplay is outlined in a recent study using data from the Adolescent Brain Cognitive Development (ABCD) Study [45]. This study found that functional brain connectivity patterns in early adolescence, associated with accelerated maturation, can predict substance use initiation and are linked to pollution exposure. Researchers examined whether resting-state functional connectivity (rsFC), measured longitudinally from pre-adolescence (ages 9–10) to early adolescence (ages 11–12), could predict future substance use initiation. They then tested whether these connectivity patterns were influenced by earlier environmental exposures, particularly neighborhood pollution and socioeconomic factors, in an independent subsample. The resting state functional connectivity (rsFC) pattern associated with substance use initiation was linked to accelerated maturation and predicted by higher exposure to pollution, even after adjusting for family socioeconomic factors. Expanding on these previous findings, our study examines the associations between rsFC within the sensory-motor network (mouth and hand regions) and key social, psychological, and behavioral factors, including baseline and past socioeconomic status (SES), trauma exposure, family conflict, impulsivity, major depressive disorder (MDD), and future substance use.

Based on prior research suggesting that sensory-motor connectivity reflects neurodevelopmental processes and is shaped by environmental and psychosocial factors, we propose the following hypotheses. First, regarding the association between socioeconomic disadvantage and sensory-motor connectivity, we hypothesize that lower socioeconomic status (SES) will be associated with altered resting-state functional connectivity (rsFC) within the sensory-motor network, particularly in the hand and mouth regions. Furthermore, these alterations will manifest as either hyperconnectivity within the sensory-motor network, indicating stronger intra-network connectivity but weaker integration with association cortices, or hypoconnectivity, reflecting overall reduced connectivity. The direction of these alterations is expected to depend on the specific SES-related exposures. Second, regarding the link between early-life stress, trauma, and sensory-motor connectivity, we hypothesize that greater exposure to early-life stress, including trauma and family conflict, will be associated with sensory-motor connectivity patterns indicative of neural adaptations to stress. Specifically, individuals with higher trauma exposure are expected to exhibit increased connectivity within the sensory-motor network, potentially reflecting heightened vigilance or hyperarousal, alongside reduced connectivity with higher-order cognitive control regions, suggesting impaired top-down regulation. Third, in relation to sensory-motor connectivity and behavioral regulation, we hypothesize that weaker integration of sensory-motor networks with cognitive control regions will be associated with higher impulsivity and reduced self-regulation, increasing susceptibility to maladaptive behaviors. Additionally, stronger intra-network connectivity but weaker inter-network connectivity may indicate a reliance on reflexive motor behaviors rather than goal-directed actions, potentially contributing to behavioral dysregulation. Fourth, regarding sensory-motor connectivity and mental health outcomes, we hypothesize that individuals with altered sensory-motor connectivity, particularly those showing weaker connectivity with executive function regions, will have a higher likelihood of major depressive disorder (MDD) symptoms. This pattern may reflect disruptions in sensorimotor processing of emotional and social stimuli, potentially contributing to difficulties in emotion regulation. Finally, in terms of the link between sensory-motor connectivity and substance use initiation, we expect that sensory-motor connectivity patterns, particularly those associated with accelerated neural maturation, will predict early substance use initiation. These connectivity patterns are anticipated to explain the relationship between early-life adversity (e.g., SES, trauma, and family conflict) and subsequent substance use, suggesting a neurodevelopmental pathway linking environmental exposures to behavioral outcomes.

By testing these hypotheses, this study aims to clarify how sensory-motor network connectivity serves as a potential biomarker of neurodevelopmental adaptation to socioeconomic and psychosocial stressors, with implications for cognitive, emotional, and behavioral health trajectories.

## 2. Methods

### 2.1. Study Design and Sample

This study utilized data from the baseline wave of the Adolescent Brain Cognitive Development (ABCD) [46-53] Study, a large, national longitudinal study of children in the United States. The ABCD Study recruited children aged 9-10 years old and has followed them through adolescence to assess various neurodevelopmental, cognitive, behavioral, and environmental measures. The current study focuses on cross-sectional associations between resting-state functional connectivity (rsFC) in the sensory-motor network and key social, psychological, and behavioral factors at baseline, while marijuana use was assessed prospectively over the next four years.

### 2.2. Analytical Sample

Our analysis included all ABCD participants, regardless of race/ethnicity, sex, SES, or the availability of study variables. The sample consisted of 11,878 children aged 9–10 at baseline, irrespective of their follow-up duration. Therefore, no specific inclusion or exclusion criteria were applied.

### 2.3. Neuroimaging Measures

Resting-state functional connectivity was assessed using functional magnetic resonance imaging (fMRI) collected at baseline. The primary regions of interest were the sensory-motor network, specifically connectivity between the mouth and hand regions. Preprocessing of fMRI data included motion correction, spatial normalization, and filtering to remove artifacts. Connectivity values were extracted using standard pipeline methods provided by the ABCD Study [54-63], quantifying the strength of rsFC between the sensory-motor hand and mouth regions [64,65]. The ABCD study implements rigorous harmonization protocols to ensure consistency and comparability of MRI data across its multiple study sites. Given the use of different MRI scanners and varying acquisition conditions, the study employs standardized imaging protocols, centralized quality control procedures, and advanced post-processing techniques to minimize site-specific variability. Harmonization efforts include scanner calibration, gradient distortion correction, and signal normalization, ensuring that structural and functional MRI data are reliable for large-scale analyses. These procedures enhance the validity of neuroimaging findings and enable robust cross-site comparisons in the study of adolescent brain development [54-63].

### 2.4. Socioeconomic Status (SES)

Socioeconomic status was measured using both family income and parental education, which were reported by caregivers at baseline. Family income was categorized into standardized brackets ranging from low-income households (<$25,000 annually) to high-income households (>$200,000 annually). Parental education was assessed as the highest level of education attained by either parent, ranging from less than high school to graduate or professional degrees.

### 2.5. Early-Life Adversity

Early-life adversity was examined using two key indicators: trauma exposure [66] and family conflict [67,68]. Trauma exposure was measured using caregiver reports on whether the child had experienced potentially traumatic events, including physical abuse, neglect, household substance use, or exposure to domestic violence. Family conflict was assessed using validated self-report measures from the caregiver, capturing levels of household discord, arguments, and overall relational instability.

### 2.6. Psychological and Behavioral Factors

Impulsivity was assessed using the UPPS-P [69-71], also known as Impulsive Behavior Scale [72], which evaluates dimensions of impulsive traits, including negative urgency, sensation seeking, and lack of premeditation. Higher scores on this measure indicate greater impulsivity, which has been associated with risk-taking behaviors in adolescence [73]. Major depressive disorder (MDD) was measured using the Kiddie Schedule for Affective Disorders and Schizophrenia (K-SADS) [74], a structured diagnostic interview that identifies clinical and subclinical symptoms of depression in children (yes/no endorsement of diagnosis as a binary variable).

### 2.7. Puberty Status

Puberty status was a dichotomous variable indicating the presence or absence of any signs of puberty at baseline (9–10 years old). This classification was based on self-reported physical changes, including Tanner staging [75], which assesses the development of secondary sexual characteristics. Puberty status was separately determined for males and females, incorporating indicators such as breast development and menarche for females and genital development and facial hair growth for males. In ABCD, both children and their parents provided reports on these changes, ensuring a more comprehensive assessment of pubertal status at baseline [76,77].

Cognitive function was assessed using multiple neurocognitive tasks from the NIH Toolbox, including measures of working memory, executive function, and verbal comprehension [78,79]. These scores were standardized, with higher values indicating better cognitive performance.

### 2.8. Substance Use Outcomes

Marijuana and tobacco use were assessed prospectively over the four years following baseline through self-reported substance use surveys administered annually. Marijuana and tobacco use during the follow-up period was quantified as binary (yes/no) outcomes. For tobacco use, the primary outcome for marijuana use was any self-reported use by ages 13–14, capturing early initiation. Since no participants in this age group had reported marijuana use at baseline, this measure allowed us to examine the predictive value of baseline rsFC and psychosocial factors for future substance use risk. more than 100 children had reported at least a puff at baseline; however, this was not considered an outcome in our analysis. Instead, we focused on subsequent tobacco use, defined as having more than a puff, among those who had not engaged in any use at baseline. In addition to assessing substance use behaviors, we also examined substance use norms and attitudes, which reflect an individual’s perceptions and beliefs about the acceptability and prevalence of substance use within their social environment. These measures provided further context for understanding the social and cognitive factors that may contribute to early substance use initiation. The substance use measures of the ABCD are described elsewhere [53].

### 2.9. Statistical Analysis

Bivariate Pearson correlations were conducted to examine associations between resting-state functional connectivity (rsFC) in the sensory-motor mouth and hand regions and key independent variables, including SES, trauma exposure, family conflict, impulsivity, major depressive disorder (MDD), cognitive function, and subsequent marijuana use. All statistical analyses were performed using Stata, with significance set at p < 0.05. Missing data were not imputed; however, cases with missing data were included in other analyses where applicable.

### 2.10. Ethical Considerations

The ABCD Study was approved by institutional review boards at participating research sites including but not limited to University of California San Diego (UCSD). Written informed consent was obtained from caregivers, and assent was obtained from all participating children. Data used in this study were de-identified and publicly available, ensuring adherence to ethical guidelines for research involving human participants.

## 3. Results

Table 1 shows that the resting-state functional connectivity (rsFC) between the sensory-motor networks of mouth and hand (SMN-MH) was significantly associated with multiple demographic, socioeconomic, mental health, substance use, and cognitive variables.

### 3.1. Demographic Correlates:

Age was positively correlated with rsFC between the sensory-motor networks of mouth and hand (r = 0.03, p < 0.05), while sex (male) was not significantly associated with rsFC between the sensory-motor networks of mouth and hand. Among racial/ethnic groups, rsFC between the sensory-motor networks of mouth and hand was higher in Black individuals (r = 0.14, p < 0.05) and Latino individuals (r = 0.08, p < 0.05) but was lower in White individuals (r = −0.15, p < 0.05).

### 3.2. Socioeconomic Correlates:

Higher parental education (r = −0.11, p < 0.05) and family income (r = −0.14, p < 0.05) were associated with lower rsFC between the sensory-motor networks of mouth and hand. In contrast, financial difficulty was positively associated with rsFC between the sensory-motor networks of mouth and hand (r = 0.08, p < 0.05).

### 3.3. Psychosocial Correlates:

rsFC between the sensory-motor networks of mouth and hand was positively correlated with past major depressive disorder (MDD) (r = 0.03, p < 0.05), puberty status (r = 0.03, p < 0.05), trauma exposure (r = 0.02, p < 0.05), and positive urgency (r = 0.03, p < 0.05).

### 3.4. Substance Use Correlates:

rsFC between the sensory-motor networks of mouth and hand showed a small but significant correlation with marijuana use (r = 0.02, p < 0.05). We also found a correlation between rsFC in this network and substance use attitudes (r =− 0.02, p < 0.05). However, rsFC between the sensory-motor networks of mouth and hand was not significantly correlated with tobacco use or substance use norms.

### 3.5. Cognitive Correlates:

Several cognitive variables were significantly associated with rsFC between the sensory-motor networks of mouth and hand. Notably, higher cognitive abilities were associated with lower rsFC between the sensory-motor networks of mouth and hand. Specifically, rsFC between the sensory-motor networks of mouth and hand was negatively correlated with picture vocabulary (r = −0.10, p < 0.05), list learning (r = −0.05, p < 0.05), card sorting (r = −0.05, p < 0.05), pattern recognition (r = −0.03, p < 0.05), picture reasoning (r = −0.03, p < 0.05), reading ability (r = −0.05, p < 0.05), fluid cognition (r = −0.06, p < 0.05), crystallized cognition (r = −0.09, p < 0.05), and total cognitive composite score (r = −0.09, p < 0.05).

## 4. Discussion

### 4.1. Aim and Hypotheses

This study aimed to explore the potential associations between rsFC in the sensory-motor network (mouth and hand regions) and key social, psychological, and behavioral factors in adolescents. Given emerging evidence suggesting that sensory-motor connectivity may serve as a proxy for cortical and brain development, we hypothesized that rsFC between the sensory-motor mouth and hand regions might be associated with SES, trauma exposure, family conflict, cognitive function, impulsivity, and MDD at baseline. Additionally, we hypothesized that stronger rsFC in this network might predict an increased risk of future substance use, potentially reflecting neurodevelopmental pathways linking early adversity to later behavioral health outcomes. Some research suggests that rsFC between the sensory-motor network mouth and hand regions may reflect the extent to which the sensory-motor network has integrated with emotional regulation and behavioral responses.

### 4.2. Summary of Findings

Our findings suggest that stronger connectivity between the sensory-motor hand and mouth regions are associated with multiple psychosocial stressors. Specifically, lower SES, higher childhood trauma exposure, and greater family conflict were all correlated with increased sensory-motor connectivity. Additionally, heightened rsFC in this network appeared to be linked to higher impulsivity and a greater likelihood of MDD at baseline. Lower cognitive function across all domains examined was also associated with stronger rsFC in this network. Longitudinal analyses further suggested that baseline rsFC in this network might predict future risk of future substance use, pointing to a possible neural mechanism through which early-life adversity could contribute to behavioral health vulnerabilities.

### 4.3. Sensory-Motor Connectivity and SES

We observed that both baseline and past SES were inversely associated with sensory-motor connectivity, suggesting that adolescents from lower-SES backgrounds may exhibit stronger rsFC within this network. Socioeconomic disparities have been linked to differences in brain maturation, myelination, and functional integration [80-87], possibly due to variations in environmental enrichment, access to structured motor activities, and exposure to chronic stress [88]. This heightened connectivity may reflect altered neurodevelopmental trajectories in low-SES children, which could have implications for behavioral regulation and stress responsivity.

### 4.4. Trauma and Family Conflict

Higher trauma exposure and greater family conflict were both associated with stronger connectivity in the sensory-motor hand-mouth network, suggesting that early-life adversity may shape the functional organization of this system. One possible explanation is that trauma-related hyperarousal may contribute to heightened sensorimotor integration, potentially as an adaptive mechanism for increased vigilance to environmental threats. Similarly, chronic family conflict may lead to persistent engagement of sensory-motor circuits, reinforcing stress responses and motor reactivity patterns. These findings align with previous research suggesting that trauma exposure may be linked to altered sensorimotor processing and increased somatic symptoms in psychiatric conditions [89-92].

### 4.5. Impulsivity and MDD

Adolescents with greater impulsivity and MDD exhibited stronger connectivity within the sensory-motor network, suggesting that these traits share neural substrates related to sensorimotor regulation. Impulsivity has been associated with weaker top-down cognitive control over motor responses [93-95], which may explain the observed relationship with increased rsFC in sensory-motor regions. Similarly, MDD is often characterized by psychomotor disturbances, somatic symptoms, and altered bodily awareness, [96-99] all of which may be reflected in atypical connectivity in motor-related brain regions. These results may represent a neural overlap between affective and behavioral dysregulation, though further research is needed to confirm this in clinical populations.

### 4.6. Sensory-Motor Connectivity and Future Substance Use

One of the most notable findings was the longitudinal association between sensory-motor connectivity and future substance use, suggesting that early connectivity patterns might serve as markers of risk. Stronger rsFC in this network may indicate greater habitual motor activation, which could predispose individuals to compulsive or automatic behaviors, including substance-seeking actions. Given that many substances—such as nicotine and alcohol—are consumed through hand-to-mouth behaviors, increased connectivity in this network may reflect neural reinforcement of habitual substance use over time. These findings provide tentative support for the idea that neurodevelopmental trajectories in sensorimotor circuits may contribute to risk-taking behaviors in adolescence.

### 4.7. Sensory-Motor Connectivity and MDD

Resting-state functional connectivity (rsFC) in the sensory-motor network was associated with higher levels of trauma and MDD, both of which are established risk factors for substance use initiation. However, what remains to be explored is whether heightened rsFC in this network serves as a predictor of substance use through its links to these traits. Given that MDD often co-occurs with increased physiological reactivity and altered sensory processing, changes in rsFC may reflect neural mechanisms that contribute to both emotional dysregulation and maladaptive coping behaviors, such as substance use. Additionally, trauma-related hyperconnectivity in sensory-motor regions may indicate heightened stress sensitivity, leading individuals to self-medicate distress through substance use. Future research should investigate whether rsFC in this network mediates the relationship between trauma, MDD, and early substance use initiation, potentially identifying neural pathways that underlie these behavioral vulnerabilities.

### 4.8. Sensory-Motor Connectivity and impulsivity

The sensory-motor network rsFC was also associated with higher levels of trauma and MDD, both of which are linked to impulsivity and increased risk of substance use initiation. What remains to be examined is whether heightened rsFC in this network acts as a predictor of substance use through its association with impulsivity and trauma-related neurobiological changes. Given that impulsivity is a key trait driving early experimentation with substances, and that trauma can amplify impulsive decision-making, it is plausible that rsFC alterations in sensory-motor regions contribute to these risk pathways. Increased connectivity in these areas may reflect heightened reactivity to external stimuli, impaired inhibitory control, or dysregulated motor responses, all of which could facilitate engagement in risky behaviors, including substance use. Future studies should explore whether sensory-motor rsFC mediates the link between trauma, impulsivity, and substance use initiation, helping to identify potential neural targets for prevention efforts.

### 4.9. Inverse Correlation Between rsFC and Cognitive Function

Stronger rsFC in the sensory-motor network was inversely associated with cognitive function, suggesting a potential trade-off between heightened sensorimotor integration and higher-order cognitive processing. Elevated connectivity in this network may reflect increased engagement in automatic, habitual responses at the expense of executive control processes critical for learning, problem-solving, and decision-making. This pattern aligns with prior research indicating that excessive sensorimotor activation may interfere with cognitive flexibility, working memory, and attentional control [100]. One possible explanation is that heightened connectivity in these regions could reflect neural resources being allocated toward sensorimotor processing rather than cognitive regulation. Alternatively, it may signal underlying neurodevelopmental differences that prioritize motor-related processing over abstract reasoning and goal-directed cognition. These findings highlight the need for further investigation into how neurodevelopmental trajectories in the sensory-motor network influence cognitive performance across adolescence.

Future research may explore the implications of this network connectivity for ADHD [101], particularly in understanding how alterations in sensory-motor connectivity contribute to core symptoms of the disorder. Our findings suggest that heightened rsFC in the sensory-motor network is associated with traits such as impulsivity and cognitive difficulties, both of which are hallmark features of ADHD. This supports the idea that sensory-motor hyperconnectivity may reflect underlying neural mechanisms that contribute to the balance—or trade-off—between motor function and cognitive control. Children with ADHD often exhibit difficulties in executive function, attention regulation, and impulse control, which may be partially explained by disruptions in the coordination between sensory-motor and cognitive networks.

Additionally, the observed associations between trauma, MDD, and altered rsFC may provide insight into the high comorbidity between ADHD and affective disorders. If heightened sensory-motor connectivity is linked to both impulsivity and emotion dysregulation, this could explain why children with ADHD are more likely to engage in risk-taking behaviors, including early substance use. More broadly, our findings suggest that disruptions in these neural networks may contribute to attention problems beyond ADHD, reinforcing the importance of studying the interactions between motor, cognitive, and affective systems. Future studies should investigate whether sensory-motor rsFC mediates the relationship between early-life stress, attention difficulties, and behavioral dysregulation, potentially offering new targets for intervention in children at risk for ADHD and related conditions.

### 4.10. Implications

These findings, if replicated in other datasets and settings, may have important implications for understanding the potential neural mechanisms underlying behavioral and psychiatric vulnerabilities in adolescents. First, the sensory-motor network is crucial for integrating external sensory information with motor output, forming the foundation for physical movement, habitual behaviors, and embodied cognition. During typical development, connectivity within sensorimotor regions strengthens, while connections to higher-order cognitive regions, such as the prefrontal cortex, may become more refined. This shift is thought to allow for increased behavioral regulation, goal-directed actions, and cognitive flexibility. However, disruptions in this process—whether due to socioeconomic disadvantage, trauma, or psychiatric vulnerabilities—may contribute to overactive sensory-motor circuits, reinforcing automatic, impulsive, or maladaptive behaviors [102]. Second, they highlight the possibility that early-life adversity influences sensory-motor connectivity, emphasizing the importance of interventions that mitigate the effects of stress and trauma on neurodevelopment. Third, the potential link between sensory-motor rsFC and future substance use risk suggests that early identification of at-risk children through neuroimaging markers could help inform targeted prevention efforts. Additionally, these results may provide insight into how impulsivity and depression share neural pathways related to motor regulation, opening new avenues for integrated treatment approaches that address both affective and behavioral dysregulation.

### 4.11. Limitations

Despite its strengths, this study has several limitations. First, as a cross-sectional analysis of resting-state functional connectivity, it cannot establish causality. Future research should incorporate longitudinal neuroimaging to track how these connectivity patterns evolve over time. Second, although the ABCD dataset provides a large and diverse sample, it remains subject to sociocultural (e.g., parenting) and environmental (e.g., neighborhood and school conditions) confounds that may influence both brain function and behavioral outcomes [32]. Third, many of the behavioral and health outcomes examined, such as substance use behaviors, are shaped by multiple external factors not fully accounted for in this study [103]. For instance, peer influence and family environment significantly impact adolescent substance use [104-106], while parental depression increases the risk of MDD in offspring [107-109]. Likewise, cognitive function is influenced by various environmental factors, including nutrition [110], air pollution [111], and other social determinants of health [112]. In addition, we did not control study site or region of the country. We also did not run multi-variable analysis that control for age, sex, or SES. Finally, while we focused on sensorimotor hand-mouth connectivity, other large brain networks also contribute to behavioral and health outcomes such as substance use [113], cognition [114], impulsivity [115], and MDD [116], and should be explored in future research.

## 5. Conclusion

This study provides evidence that rsFC between the sensory-motor hand and mouth regions may be associated with SES, trauma exposure, family conflict, impulsivity, lower cognitive function, and depression, and might predict future marijuana use in adolescents. These findings suggest that sensory-motor connectivity could reflect both environmental and neurodevelopmental influences, potentially serving as an early marker of risk for behavioral and psychiatric vulnerabilities. By further exploring these neural pathways, future research may help inform preventative interventions that promote resilience in at-risk children, ultimately contributing to more effective strategies for addressing socioeconomic disparities, mental health challenges, and substance use prevention.

## Figures and Tables

**Table 1.** Bivariate correlations (Pearson test) between study variables

	1	2	3	4	5	6	7	8	9	10	11	12	13	14	15	16	17	18	19	20	21	22	23	24	25	26	27	28	29	30	31
rsFC	1.00																														
Age	0.03	1.00																													
Sex (Male)	−0.03	0.02	1.00																												
Race/Ethnicity (White)	−0.15	0.02	0.02	1.00																											
Race/Ethnicity (Black)	0.14	0.00	−0.02	−0.44	1.00																										
Race/Ethnicity (Latino)	0.08	−0.02	0.00	−0.52	−0.21	1.00																									
Race/Ethnicity (Asian)	−0.01	0.02	−0.01	−0.15	−0.06	−0.07	1.00																								
Race/Ethnicity (Other)	0.00	−0.01	0.00	−0.36	−0.14	−0.17	−0.05	1.00																							
Parental Education	−0.11	0.02	0.00	0.36	−0.22	−0.30	0.11	0.02	1.00																						
Family Income	−0.14	0.04	0.01	0.43	−0.37	−0.22	0.06	−0.02	0.62	1.00																					
Financial Difficulty (n)	0.08	−0.01	0.01	−0.20	0.21	0.05	−0.05	0.04	−0.28	−0.42	1.00																				
Family Conflict	0.03	0.00	0.04	−0.04	0.07	−0.02	−0.02	0.03	−0.06	−0.10	0.19	1.00																			
MDD (Past)	0.03	0.02	0.02	−0.04	0.05	0.01	−0.01	0.00	−0.04	−0.04	0.05	0.01	1.00																		
Puberty (Any)	0.03	0.11	−0.08	−0.08	0.09	0.01	0.01	0.01	−0.06	−0.07	0.04	0.01	0.04	1.00																	
Trauma (Any)	0.02	0.01	−0.02	−0.05	0.07	−0.01	−0.04	0.04	−0.07	−0.12	0.16	0.12	0.03	0.05	1.00																
Positive Urgency	0.03	−0.03	0.08	−0.11	0.09	0.03	−0.02	0.03	−0.13	−0.14	0.11	0.05	0.09	0.06	0.05	1.00															
Substance Use (Tobacco)	0.00	0.07	−0.01	−0.01	−0.01	0.02	−0.03	0.03	−0.04	−0.05	0.04	0.04	0.03	0.05	0.06	0.04	1.00														
Substance Use (Marijuana)	0.02	0.08	0.00	−0.02	0.01	0.02	−0.02	0.00	−0.06	−0.07	0.06	0.03	0.03	0.04	0.05	0.04	0.45	1.00													
Substance Use Knowledge	0.00	0.02	−0.03	0.03	−0.05	0.02	0.02	−0.05	0.06	0.03	−0.03	−0.02	−0.10	−0.03	−0.05	−0.09	−0.09	−0.07	1.00												
Substance Use Norms (Peers)	−0.01	0.05	−0.02	0.04	−0.07	0.01	0.02	−0.02	0.09	0.06	−0.07	−0.01	−0.04	0.00	−0.05	−0.09	−0.04	−0.03	0.98	1.00											
Substance Use Attitudes	−0.02	0.03	−0.05	0.05	−0.04	−0.03	0.00	−0.01	0.06	0.07	−0.05	−0.04	−0.01	−0.01	−0.02	−0.08	−0.03	−0.03	0.21	0.22	1.00										
Substance Use (Any)	−0.04	0.09	0.05	0.10	−0.11	−0.03	−0.02	0.01	0.10	0.10	−0.02	0.01	0.01	0.05	0.04	0.05	0.42	0.35	−0.12	−0.07	−0.07	1.00									
Cognition Picture Vocabulary	−0.10	0.01	0.03	0.33	−0.29	−0.17	0.05	0.01	0.39	0.38	−0.19	−0.07	−0.04	−0.05	−0.05	−0.16	−0.02	−0.01	0.04	0.07	0.07	0.11	1.00								
Cognition Flanker	−0.02	−0.02	0.03	0.07	−0.12	−0.02	0.07	0.02	0.15	0.16	−0.07	−0.03	−0.02	−0.01	−0.02	−0.06	0.00	0.00	−0.02	0.02	0.02	0.05	0.20	1.00							
Cognition List	−0.05	0.03	0.03	0.21	−0.21	−0.09	0.04	0.00	0.29	0.29	−0.13	−0.05	−0.04	−0.04	−0.04	−0.12	−0.03	−0.01	0.05	0.07	0.04	0.05	0.38	0.25	1.00						
Cognition Card Sorting	−0.05	0.00	−0.04	0.14	−0.16	−0.06	0.06	0.01	0.19	0.20	−0.11	−0.03	−0.03	−0.01	−0.03	−0.10	−0.01	−0.01	0.01	0.03	0.03	0.04	0.23	0.43	0.26	1.00					
Cognition Pattern	−0.03	0.09	−0.07	0.08	−0.12	−0.02	0.06	0.00	0.11	0.12	−0.07	−0.03	−0.02	0.00	−0.03	−0.09	0.00	0.00	−0.01	0.01	0.02	0.03	0.16	0.35	0.19	0.41	1.00				
Cognition Picture	−0.03	0.00	−0.07	0.15	−0.19	−0.03	0.05	0.00	0.18	0.19	−0.11	−0.04	−0.03	−0.02	−0.03	−0.08	−0.03	−0.04	0.04	0.04	0.02	0.03	0.23	0.18	0.33	0.24	0.18	1.00			
Cognition Reading	−0.05	0.01	0.01	0.16	−0.20	−0.08	0.10	0.03	0.30	0.29	−0.15	−0.05	−0.03	−0.01	−0.05	−0.13	−0.01	−0.01	0.02	0.06	0.05	0.08	0.50	0.21	0.36	0.22	0.16	0.21	1.00		
Cognition Fluid	−0.06	0.04	−0.04	0.19	−0.24	−0.06	0.08	0.01	0.26	0.28	−0.14	−0.05	−0.04	−0.03	−0.05	−0.14	−0.02	−0.01	0.02	0.05	0.04	0.06	0.35	0.64	0.59	0.71	0.72	0.58	0.35	1.00	
Cognition Crystalized	−0.09	0.01	0.02	0.28	−0.28	−0.14	0.08	0.02	0.40	0.39	−0.20	−0.07	−0.05	−0.03	−0.06	−0.17	−0.02	−0.01	0.03	0.07	0.07	0.11	0.85	0.24	0.43	0.26	0.18	0.26	0.88	0.40	1.00
Cognition Total Composite	−0.09	0.03	−0.01	0.28	−0.31	−0.12	0.10	0.02	0.40	0.40	−0.20	−0.07	−0.05	−0.03	−0.06	−0.18	−0.02	−0.01	0.03	0.07	0.07	0.10	0.72	0.52	0.61	0.57	0.53	0.49	0.74	0.83	0.85

**Note**: rsFC: rsFC between the sensory-motor network mouth and hand regions. Numbers equal or larger than 0.02 are statistically significant at p <0.05.
